# Localization of Sphingolipid Enriched Plasma Membrane Regions and Long-Chain Base Composition during Mature-Fruit Abscission in Olive

**DOI:** 10.3389/fpls.2017.01138

**Published:** 2017-06-29

**Authors:** Maria C. Parra-Lobato, Miguel A. Paredes, Juana Labrador, Mariana Saucedo-García, Marina Gavilanes-Ruiz, Maria C. Gomez-Jimenez

**Affiliations:** ^1^Department of Plant Physiology, University of ExtremaduraBadajoz, Spain; ^2^Institute of Agricultural Sciences, Autonomous University of the State of HidalgoTulancingo, Mexico; ^3^Departamento de Bioquímica, Facultad de Química, Universidad Nacional Autónoma de MéxicoMexico City, Mexico

**Keywords:** abscission, fruit, olive, sphingolipid, sterol, vesicle trafficking

## Abstract

Sphingolipids, found in membranes of eukaryotic cells, have been demonstrated to carry out functions in various processes in plant cells. However, the roles of these lipids in fruit abscission remain to be determined in plants. Biochemical and fluorescence microscopy imaging approach has been adopted to investigate the accumulation and distribution of sphingolipids during mature-fruit abscission in olive (*Olea europaea* L. cv. Picual). Here, a lipid-content analysis in live protoplasts of the olive abscission zone (AZ) was made with fluorescent dyes and lipid analogs, particularly plasma membrane sphingolipid-enriched domains, and their dynamics were investigated in relation to the timing of mature-fruit abscission. In olive AZ cells, the measured proportion of both polar lipids and sphingolipids increased as well as endocytosis was stimulated during mature-fruit abscission. Likewise, mature-fruit abscission resulted in quantitative and qualitative changes in sphingolipid long-chain bases (LCBs) in the olive AZ. The total LCB increase was due essentially to the increase of t18:1(8*E*) LCBs, suggesting that C-4 hydroxylation and Δ8 desaturation with a preference for (*E*)-isomer formation were quantitatively the most important sphingolipids in olive AZ during abscission. However, our results also showed a specific association between the dihydroxylated LCB sphinganine (d18:0) and the mature-fruit abscission. These results indicate a clear correlation between the sphingolipid composition and mature-fruit abscission. Moreover, measurements of endogenous sterol levels in the olive AZ revealed that it accumulated sitosterol and campesterol with a concomitant decrease in cycloartenol during abscission. In addition, underlying the distinct sterol composition of AZ during abscission, genes for key biosynthetic enzymes for sterol synthesis, for obtusifoliol 14α-demethylase (CYP51) and C-24 sterol methyltransferase2 (SMT2), were up-regulated during mature-fruit abscission, in parallel to the increase in sitosterol content. The differences found in AZ lipid content and the relationships established between LCB and sterol composition, offer new insights about sphingolipids and sterols in abscission.

## Introduction

A diverse set of lipids metabolically and structurally related, sphingolipids have many functions related to signaling as well as cell structure (reviewed in Huwiler et al., 2000; Hannun and Obeid, 2002; Pata et al., 2010; Markham et al., 2013; Pyne et al., 2014; Luttgeharm et al., 2016; Michaelson et al., 2016; Kraft, 2017). The regulation of the metabolism of these lipids is complex, involving multiple enzymatic pathways that mediate the interconversion of virtually every sphingolipid species, which are localized in distinct subcellular compartments. Recent knowledge of the functions of sphingolipids in plants has revealed their critical role both in physiological or stress situations (Michaelson et al., 2016). Part of the vital necessity of the plant to possess sphingolipids can be explained in terms of the competence of these lipids to form the lipid matrix of the membranes and hence, to their contribution of their functions. The amphipatic structural backbone of complex sphingolipids is ideal to form lipid bilayers. This backbone is composed of a long-chain base (LCB) that amidates a fatty acid and binds a polar head formed by a carbohydrate and, optionally, a phosphate group (Chen et al., 2009).

Sphingolipid synthesis begins in the ER with the condensation of serine and palmitoyl-CoA to produce ketosphinganine. When this product is reduced, it results in a 1,3-dihydroxy LCB, also called sphinganine or d18:0 (18 carbons, two hydroxyl groups, and no double bonds). This LCB can be modified to originate other LCB forms. For example, the addition of a hydroxyl group in the C4 of d18:0 produces t18:0 or phytosphingosine. Another type of modification consists of a desaturation of d18:0 and t18:0 between C4 and C5 in *trans* and/or between C8 and C9 in *cis* or *trans* positions. This diversity of modifications renders nine different LCB species in plants (Markham et al., 2013). The ceramide backbone of a complex sphingolipid includes a LCB and a fatty acid (FA) linked to the LCB via an amide linkage (Chen et al., 2009). FAs length in sphingolipids can vary from 16 to 26 carbon atoms. FAs that have between 20 and 26 carbons in length are called very-long-chain fatty acids (VLCFAs). The presence of VLCFA increases the hydrophobicity of sphingolipids, a requisite to form nanodomains (Markham et al., 2011). The ceramide portion of complex sphingolipids attaches a polar head in the C1 of the LCB, giving the amphiphilic nature to the complex sphingolipids. Among the polar groups attached to plant ceramides are glucose to form the glucosylceramide class (GlcCers), or phosphoinositol to give rise to the glycosyl inositolphosphoceramides class (GIPCs). The inositol residue is substituted with additional sugar or sugar-derived residues (Chen et al., 2009). Because of the possible chemical variation in each moiety, sphingolipids can present a huge diversity. In Arabidopsis (*Arabidopsis thaliana*) leaves, GlcCers comprise ∼30% and are enriched in dihydroxy LCBs and C16 FA, while GIPCs are the most abundant, comprising 60% and are enriched in trihydroxy LCBs and VLCFA (Markham et al., 2006, 2013).

Sphingolipids are also intimately involved in endomembrane trafficking and, besides their role as building blocks of the lipid bilayer of membranes, an additional distinctive function of sphingolipids is to form special regions of the membranes in conjunction with sterols (Foster et al., 2003; Aubert et al., 2011; Simon-Plas et al., 2011; Cacas et al., 2012; Markham et al., 2013; Yang et al., 2013). Because of their size, these regions are called nanodomains, are assembled in the *trans*-Golgi network (TGN), and then are carried to the plasma membrane (Klemm et al., 2009). They cluster proteins involved mainly in signal transduction, transport, and trafficking (Foster et al., 2003; Cacas et al., 2012; Markham et al., 2013; Yang et al., 2013). It has been suggested that sterol- sphingolipid-rich nanodomains are involved in polar targeting of proteins to plant plasma membrane (Fischer et al., 2004). More recently, substantial evidence has accumulated to indicate that membrane domains play important roles in the cell signaling in plants (Raffaele et al., 2009; Boutté and Moreau, 2014; Konrad and Ott, 2015).

Free sterols are integral components of the membrane lipid bilayer that interact with phospholipids and sphingolipids since their length is similar to that of the phospholipid monolayer (Valitova et al., 2016). Plant sterols are located mainly in the plasma membrane with respect to other cellular membranes (Hartmann, 1998; Horvath and Daum, 2013; Valitova et al., 2016). Plant sterols are a mix of three main species, i.e., campesterol, stigmasterol, and β-sitosterol (the predominant one), in addition to several minor sterols which act as biosynthetic precursors of the major sterols (Schaller, 2003; Benveniste, 2004). The bulk membrane sterols constitute campesterol, stigmasterol, β-sitosterol, and (Hartmann-Bouillon and Benveniste, 1987; Valitova et al., 2016), with campesterol being a precursor of brassinosteroids as well (Fujioka and Yokota, 2003; Valitova et al., 2016). Being key cell-membrane components, sterols are key for the growth of plants, and modulate biophysical features. Therefore, the compositional changes of sterols affect not only permeability and membrane fluidity (Roche et al., 2008; Grosjean et al., 2015) but in addition modulate the action of membrane-bound proteins (Carruthers and Melchoir, 1986; Cooke and Burden, 1990; Grandmougin-Ferjani et al., 1997) as well as adaptive reactions of plants to various kinds of stress, whether abiotic and biotic (Posé et al., 2009; Kopischke et al., 2013; Senthil-Kumar et al., 2013; Kumar et al., 2015; Wagatsuma et al., 2015; Valitova et al., 2016).

The ability to visualize sphingolipids and sterols at the subcellular level is crucial to understand sphingolipid and sterol distribution and function in plant cells. In particular, different specific fluorescent lipophilic stains of the sphingolipid enriched regions in the plasma membranes of Arabidopsis leaf tissue and protoplasts were previously visualized (Blachutzik et al., 2012), indicating that the generation of protoplasts allowed better accessibility of the plasma membrane to the application of individual lipid analogs from the extracellular side. The use of protoplasts is advantageous because the unspecific fluorescence signals due to the cell wall and its components is eliminated (Blachutzik et al., 2012).

Advances in understanding the fundamental roles of sterols and sphingolipids in a wide variety of physiological or stress situations have been gained by biochemical and molecular genetic analyses of Arabidopsis mutants for genes that encode sterol metabolic and sphingolipid enzymes (Clouse, 2002; Schaller, 2003; Markham et al., 2013; González-Solís et al., 2014; Michaelson et al., 2016). However, until now, it is still unknown whether these lipids are involved in the abscission process. Many fruit-tree species undergo massive natural fruit abscission in a large number of fruit-tree species after ripening of the fruit. For example, in olive (*Olea europaea* L.), mature-fruit abscission occurs when the abscission zone (AZ), between the fruit and its pedicel, is activated. The patterns of such abscission vary among cultivars (Gomez-Jimenez et al., 2010; Parra-Lobato and Gomez-Jimenez, 2011; Gil-Amado and Gomez-Jimenez, 2012). In certain cultivars of olive (e.g., cv. Picual), events associated with fruit ripening trigger ripe-fruit abscission from the pedicel, at some 217 days after anthesis (DPA) (Gomez-Jimenez et al., 2010; Parra-Lobato and Gomez-Jimenez, 2011). Previously, we compared the AZ transcriptomes of Picual fruit at two distinct stages (abscission vs. pre-abscission) employing the technique RNA-Seq; that is, 148 Mb of sequences (443,811 sequence reads of good quality). The result was the identification of 4,728 genes differentially expressed and identified in the two samples (Gil-Amado and Gomez-Jimenez, 2013). Among the genes governing mature-fruit abscission, we have identified various genes involved in sphingolipids and sterol biosynthesis in the olive AZ (Gil-Amado and Gomez-Jimenez, 2013). However, the impact of those changes is unknown. Consequently, the purpose of this study was to further elucidate the potential involvement of sphingolipids and sterols in the abscission process. To this end, using specific fluorescent lipophilic probes, we visualized sphingolipid-enriched regions in the plasma membranes of live protoplasts from olive AZ. We also investigated sphingolipid LCB composition during mature-fruit abscission. In addition, sterol content and biosynthetic gene expression were also analyzed during abscission. These data highlight a new facet of sphingolipids and sterols in abscission.

## Materials and Methods

### Plant Material

Olive trees (*O. europaea* L. cv. ‘Picual’) 20 years old were cultivated with drip irrigation and fertigation (water carrying appropriate fertilizers) in an orchard near Badajoz (Spain). The ‘Picual’ cultivar at maturity typically undergoes massive natural abscission of the fruit (Gomez-Jimenez et al., 2010). This abscission takes place at the pedicel-fruit AZ (Gomez-Jimenez et al., 2010). Olive flowers were tagged on the day of pollination and the AZ samples were collected from olive fruits subsequently harvested at two specified stages during fruit abscission induction: fruit AZ pre-cell separation (or pre-abscission stage) and nearly complete fruit AZ cell separation (or abscission stage) (Parra-Lobato and Gomez-Jimenez, 2011; Gil-Amado and Gomez-Jimenez, 2013). Using a razor blade, we manually dissected AZ tissues from longitudinal sections into pieces measuring a maximum of 1 mm wide on each side of the abscission fracture plane (Gil-Amado and Gomez-Jimenez, 2013; Parra et al., 2013). Fruit AZ wings containing mesocarp or pedicel/ calyx-like tissues were discarded. A first group of freshly excised AZ samples at two different stages (pre-abscission and abscission) was used for protoplast isolation, and another group was immediately frozen in liquid nitrogen and stored at -80°C for analysis of LCBs and sterol content.

### Olive AZ Protoplast Isolation

The olive-fruit AZ at two different stages (pre-abscission and abscission), after being diced, were incubated 2 h at 28°C in digestion buffer in an incubation shaker (30 rpm). The buffer was composed of pectolyase (0.1% w/v; Sigma–Aldrich, Spain), cellulase (0.8% w/v; Sigma–Aldrich, Spain), polyvinylpyrolidone (0.5% w/v; Sigma–Aldrich, Spain), and bovine serum albumin (0.5% w/v; Sigma–Aldrich, Spain), plus calcium chloride (1 mM) was added. Using sorbitol, we adjusted the osmolarity to 280 milliosmol/kg and the pH to 5.6 (MES-Tris). A short centrifugation was necessary (80–100 rpm, 10 min, 4°C) following the incubation in order to separate the protoplasts from the undigested fragments of AZ.

### Dye Staining of Protoplasts

Protoplast imaging was made using a FluoView 1000 spectral confocal microscopy (Olympus, Tokyo, Japan) equipped with 405, 488, 543, and 633 nm laser lines. Fluorescence acquisition was selected to maximize fluorochrome emission using spectral detectors, in each case. About 10 protoplasts were acquired per sample, and data were compiled using FV10 4.2 software (Olympus). For the analysis of compartmentalized fluorescence, two regions were selected in each protoplast, one free-hand region circling plasma membrane, and one spherical region containing cytoplasmic endomembranes.

### Bodipy 505/515

Bodipy (4,4-difluoro-3a,4adiaza-s-indacene) was used to detect neutral lipids (apolar) (Thermofisher). A fluorophore, Bodipy 505/515 is used for labeling a broad variety of lipids, e.g., phospholipids, fatty acids, cholesteryl esters, cholesterol, and ceramides (Elle et al., 2010). All cell structures are permeated by this dye and, when the environment is either polar or non-polar, the characteristic green fluorescence appears so that primarily neutral storage lipid droplets are stained (Govender et al., 2012). Protoplasts were incubated for 30 min in the dark at a final concentration of 50 ng/ml in protoplast buffer. Its spectral properties are 505 nm excitation wavelength and 515 nm of emission wavelength.

### Nile Red

Nile red (9-(Diethylamino)-5H benzo [α] phenoxazin-5one) staining was used to detect neutral and polar lipids. This a fluorescent lipophilic dye has an emission shift from red to yellow depending on the hydrophobicity of the lipids. When excited at 515 nm, this dye gives three emission bands with centers at 636, 580, and 530 nm, i.e., polar, total, and non-polar lipids, respectively. Nile red is used to estimate lipid levels and it is widely used to assess the lipid content in human cells, fungi and microalgae. The Nile red for plant intracellular lipid detection should be used with caution because this dye reportedly interferes with chlorophyll (Laurens and Wolfrum, 2010) and has low poor penetration into the cell wall and thus into the cell. Using protoplasts avoids the unspecific staining of the cell wall and its components. In the present study, the Nile red solution (0.1 mg/ml in acetone) was added to protoplasts and incubated for 10 min. After washing once in protoplast buffer, stained protoplasts were observed by confocal laser scanning microscopy. The orange/red fluorescence of Nile red was acquired with a 585 nm long pass filter, and the mean fluorescence intensities of olive AZ protoplasts at two different stages were measured.

### Bodipy-Sphingomyelin FL C12 (BD-SM)

The amphiphilic BD-SM lipid analog [*N*-(4,4-difluoro- 5,7-dimethyl-4-bora-3a,4a-diaza-s-indacene-3-dodecanoyl) sphingosyl phosphocholine] consists of a sphingosine moiety which is linked to a fatty acid by an amide bond. In fact, sphingomyelin is linked covalently to a Bodipyfluorophor (Thermofisher). According to manufacturer information, BD-SM has the same stereochemical makeup as biologically active sphingomyelins, despite the fluorescence labeling. As mentioned above, Bodipy’s spectral properties are 505 nm excitation wavelength and 515 nm of emission wavelength. DMSO at a stock concentration (1.0 μg/μl) was used to solve the dye. The stock solution at 1% (v/v) was used in protoplast buffer to stain protoplasts for 20 min at room-temperature.

### FM4-64

The lipophilic FM4-64 dye [*N*-(3-triethylammoniumpropyl)-4-(6-(4-(diethylamino) phenyl)hexatrienyl)pyridiumdibroide] has basically a polyethylene structure. This water-soluble dye is non-toxic to live tissues according to the manufacturer (Thermofisher). Cell viability was also confirmed using trypan blue. In pure water the dye was solved to a stock solution (1 μg/μl). The staining was optimized over various experiments. An incubation time of 10–15 min was selected together with ambient temperature in protoplast buffer and a 0.5% (v/v) final concentration for the staining of olive protoplasts. The fluorophore was excited at a wavelength of 543 nm using an FM4-64 emission spectrum in the 580–650 nm range (emission maximum: 640 nm).

### Cell Viability

Trypan blue stain at 0.4% (Sigma–Aldrich, Spain) served to indicate cell viability. For this, trypan blue was solved to 1 mg/ml in 0.6 M mannitol. Cells that are non-viable absorb the dye, appearing blue, whereas viable cells and intact membranes reject the stain. The dye was applied directly to the protoplast buffer, whereupon the suspension was thoroughly mixed. For the desired black-and-white contrast (adequate for imaging), concentrations of trypan blue were used up to 20% (v/v). After 10-min incubations at ambient temperature, staining was suitable.

### Sphingolipid Analysis

Sphingolipids were analyzed from their released LCB as previously described (Markham et al., 2006). For sample standardization, C20-4-SPH (d20:1) was added as an internal standard. Sphingolipids were hydrolyzed using the method of Morrison and Hay (1970) with modifications after Cahoon and Lynch (1991) and derivatized with *o*-phthaldialdehyde. HPLC analyses were carried out using an Agilent Technologies HPLC Infitinity 1260 by reverse phase HPLC on a 2.1 mm × 150 mm Eclipse XBD-C18 Narrow-bore column (Agilent Technologies, Inc., Palo Alto, CA, United States). Elution was performed at (0.4 ml/min) with 20% solvent RA (5 mM potassium phosphate, pH 7), 80% solvent RB (100% methanol) for 7 min, increasing to 90% solvent RB by 15 min, followed by isocratic flow for 10 min before increasing to 100% solvent RB by 30 min with a 3-min 100% solvent RB wash before returning to 80% solvent RB and re-equilibrating for 2 min. Fluorescence was excited at 340 nm and detected at 455 nm. Results were analyzed and integrated using Openlab.

### Sterol Analysis

A pool of 100 mg fresh weight of AZ samples was used for each measurement, and made in three independent biological replicates. Sterols were quantified by gas chromatography-mass spectrometry as described previously (Closa et al., 2010). 5-Alpha cholestane (Sigma–Aldrich, Spain) was added as internal standard.

### Quantitative RT-PCR

Previously published RNA-Seq data (Gil-Amado and Gomez-Jimenez, 2013), were mined for sterol biosynthesis-related genes. Expression levels were obtained for genes encoding the obtusifoliol 14 alpha-demethylase (*OeCYP51*) and sterol methyltransferase (*OeSMT2*). Total RNA (2 mg) was reverse transcribed with random hexamers and Superscript III (Invitrogen), according to the manufacturer’s instructions. Purified cDNA (2 ng) was used as a template for qRT-PCR. qRT-PCR assays were performed with gene-specific primers. Primer sequences were 5′-TACCCAAAGCTTGGTAGCGTGTT-3′ (forward) and 5′-TCGACGACGGTGTGCGGGGAT-3′ (reverse) for *OeCYP51;* and 5′-AGGGCACACAACAAGAAGGC-3′ (forward) and 5′-GATATCCTTGTAACTCCTTAGCCC-3′ (reverse) for *OeSMT2*. The cDNA was amplified using a SYBRGreen-PCR Master kit (Applied Biosystems) containing an AmpliTaq Gold polymerase on an iCycler (BioRad Munich), following the protocol provided by the supplier. Samples were subjected to thermal cycling conditions of DNA polymerase activation at 94°C, 45 s at 55°C, 45 s at 72°C, and 45 s at 80°C; a final elongation step of 7 min at 72°C was performed. The melting curve was designed to increase by 0.5°C every 10 s from 62°C. The amplicon was analyzed by electrophoresis and sequenced once for confirmation of identity. qRT-PCR efficiency was estimated via a calibration dilution curve and slope calculation. Expression levels were determined as the number of cycles needed for the amplification to reach a threshold fixed in the exponential phase of the PCR (CT). The data were normalized for the quantity of the *O. europaea* ubiquitin (*OeUB*) gene (Parra-Lobato and Gomez-Jimenez, 2011; Gil-Amado and Gomez-Jimenez, 2012). In two independent experiments, duplicates were used from three biological replicates.

## Results

### Changes of Lipid Content in Olive AZ during Mature-Fruit Abscission

**FIGURE 1:**
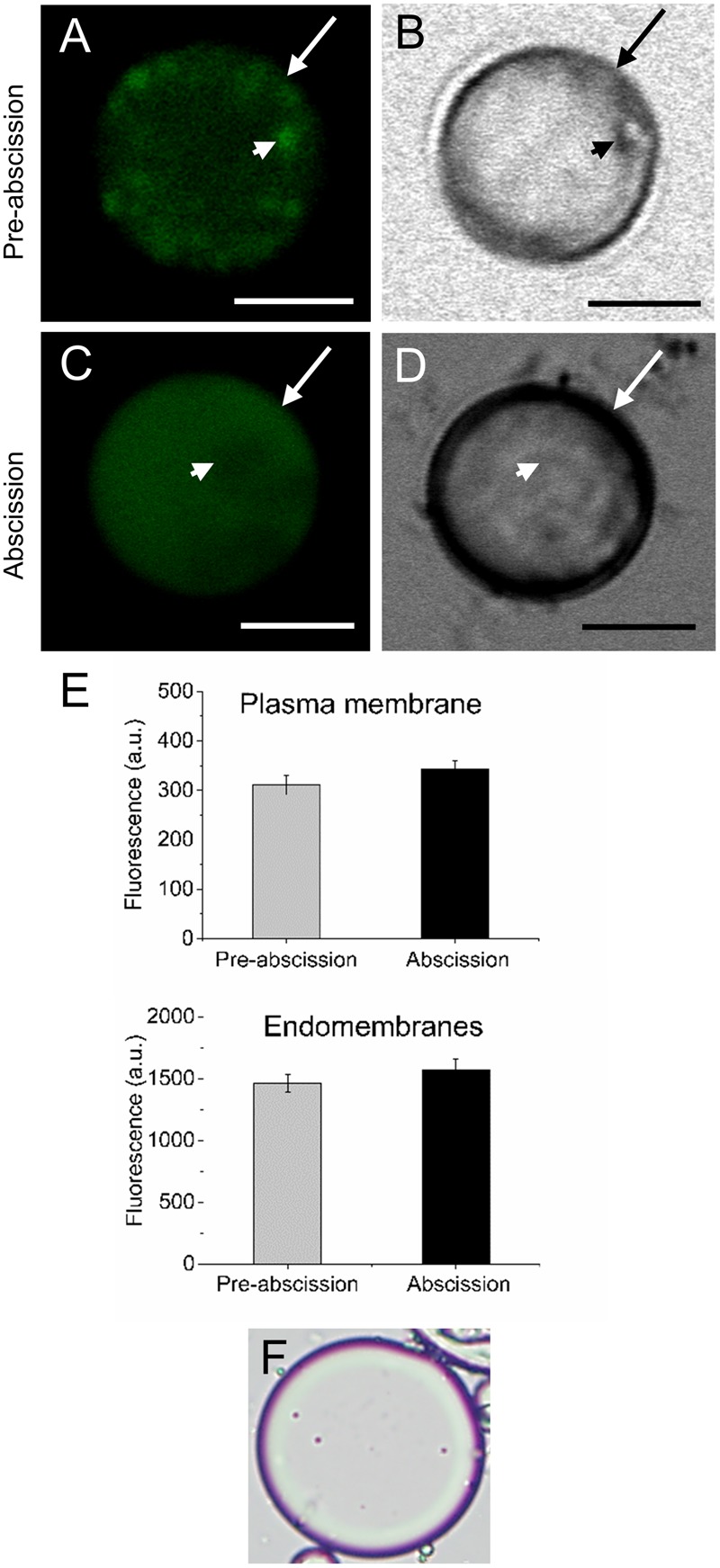
Changes in the lipid amount in live protoplasts from olive AZ during mature-fruit abscission. **(A)** Bodipy-stained protoplasts from the olive AZ at the pre-abscission stage compared to the white-light image **(B)**. **(C)** Bodipy-stained protoplasts from the olive AZ at the abscission stage compared to the white light image **(D)**. **(E)** Quantification of Bodipy fluorescence in the plasma membrane (examples marked by arrows) and the intracellular membranes (examples marked by arrowheads) of live protoplasts from olive fruit AZ at different stages during abscission. About 10 protoplasts were acquired for each sample, and data were obtained with FV10 4.2 software (Olympus). **(F)** Viability was successfully tested using trypan blue. Columns and bars indicate means ± SD, respectively, from five independent experiments. Scale bars are 5 μm. Arrow: plasma membrane. Arrowhead: endomembranes.

**FIGURE 2:**
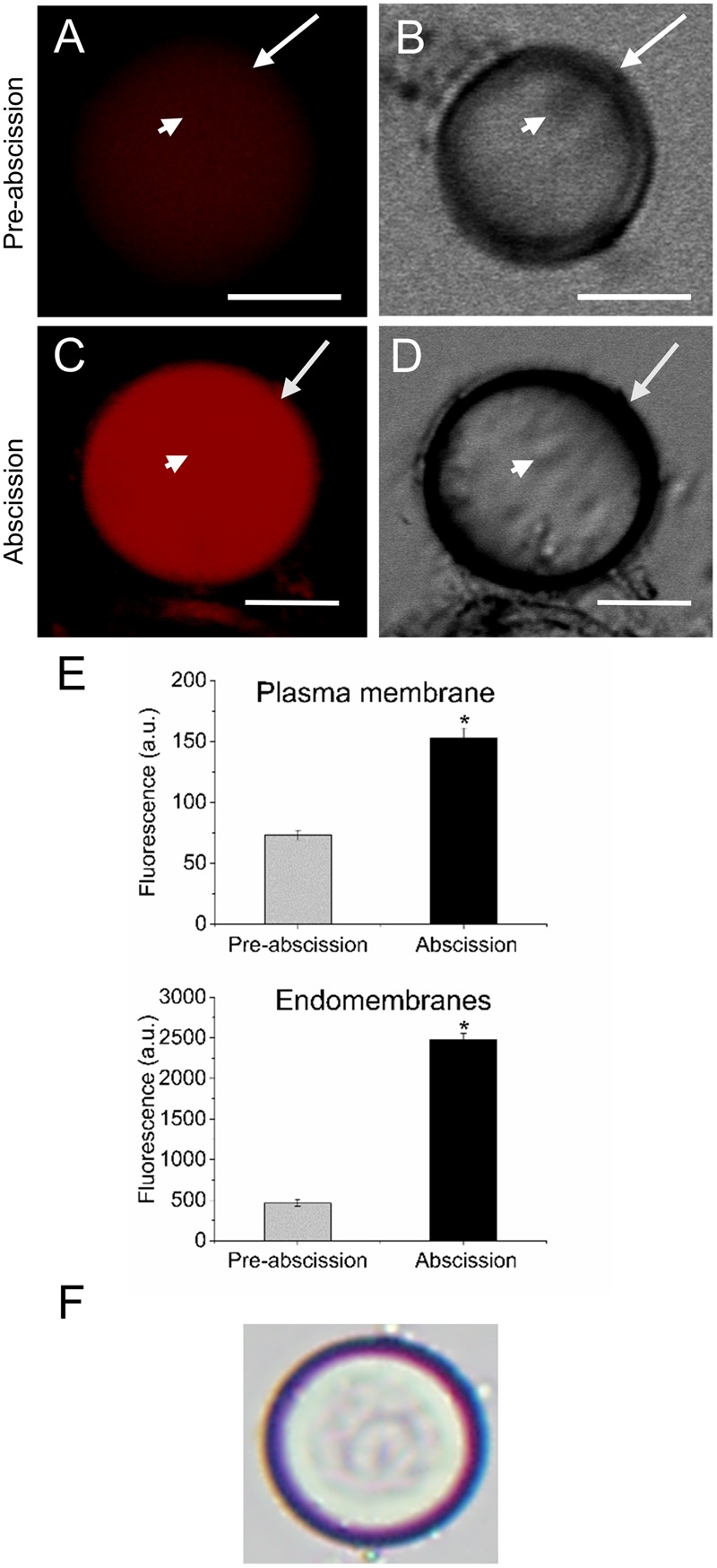
Changes on polar lipid of live protoplasts from olive AZ during mature-fruit abscission. **(A)** Nile red stained protoplasts from the olive AZ at the pre-abscission stage compared to the white-light image **(B)**. **(C)** Nile red stained protoplasts from the olive AZ at the abscission stage compared to the white-light image **(D)**. **(E)** Effect of the abscission on polar lipid content in the plasma membrane and the intracellular membranes of fruit AZ protoplasts. About 10 protoplasts were acquired for each sample, and data were compiled with FV10 4.2 software (Olympus). Columns and bars indicate means ± SD, respectively, from five independent experiments. Statistically significant differences based on unpaired Student’s *t*-test at *P* < 0.05 are denoted by an asterisk. **(F)** Shows a protoplast after trypan blue treatment; trypan blue is excluded from the cytosol of intact cells (here shown after Nile red treatment). Scale bars are 5 μm. Arrow: plasma membrane. Arrowhead: endomembranes.

According to our previous study, gene expression related to sphingolipid metabolism were up-regulated in the olive AZ during mature-fruit abscission (Gil-Amado and Gomez-Jimenez, 2013). Firstly, to elucidate whether rising levels of lipids in AZ and mature-fruit abscission induction were related events, neutral and polar lipid contents were located in live protoplasts from olive AZ in relation to the timing of mature-fruit abscission by Bodipy and Nile red staining, respectively (**Figures 1, 2**). The results showed that olive AZs at the abscission stage stained by Bodipy had lipid contents similar to those of AZs at the pre-abscission stage (**Figures 1A,C,E**). By contrast, the use of fluorescent Nile red for lipid measurement in olive AZ protoplasts revealed that abscission significantly increased the polar lipid concentration in the plasma membrane as well as in the intracellular membranes (endomembranes) (**Figures 2A,C,E**), whereas the non-polar or neutral lipid concentration measured for olive AZ at the abscission stage was close to AZ at the pre-abscission stage levels (**Supplementary Figure S1**), as was detected with the Bodipy dye.

### Visualization of Sphingolipids Enriched Plasma Membrane Regions in Olive AZ during Mature-Fruit Abscission

**FIGURE 3:**
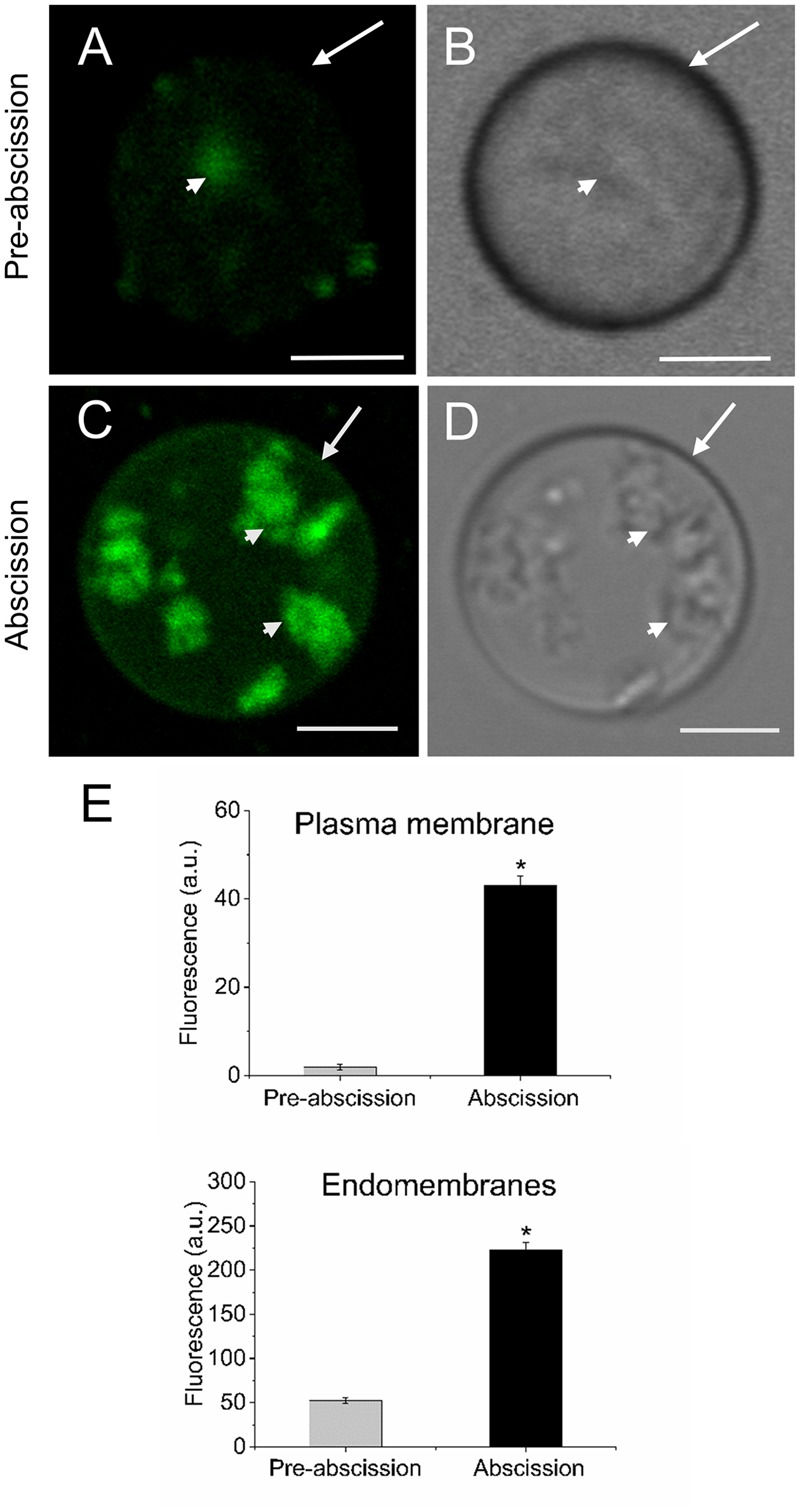
Changes in the sphingolipid content in live protoplasts from olive AZ during mature-fruit abscission. **(A)** BD-SM stained protoplast from the olive AZ at the stage pre-abscission compared to the white-light image **(B)**. **(C)** BD-SM stained protoplast from the olive AZ at the stage abscission compared to the white-light image **(D). (E)** Quantification of BD-SM fluorescence in the plasma membrane and the intracellular membranes of live protoplasts from olive fruit AZ at different stages during abscission. About 10 protoplasts were acquired for each sample, and data were obtained with FV10 4.2 software (Olympus). Columns and bars indicate means ± SD, respectively, from five independent experiments. Statistically significant differences based on unpaired Student’s *t*-test at *P* < 0.05 are denoted by asterisk. Scale bars are 5 μm. Arrow: plasma membrane. Arrowhead: endomembranes.

Next, in order to study the distribution pattern of sphingolipids in the AZ cells during mature-fruit abscission, AZ live protoplasts were stained with Bodipy Sphingomyelin FL C12 (BD-SM). BD-SM molecules incorporate into the plasma membrane and mix up with native sphingolipids (Blachutzik et al., 2012). As shown in **Figure 3**, BD-SM fluorescence increased in both the plasma membrane and the intracellular membranes of AZ protoplasts during mature-fruit abscission. Quantification showed BD-SM to be some 21-fold greater in the plasma membrane of AZ cells at the abscission stage compared with the pre-abscission stage (**Figure 3E**), but roughly a fourfold increase in the intracellular membranes of AZ cells at the abscission stage compared with the pre-abscission stage (**Figure 3E**). Therefore, there is a close relationship between high sphingolipid levels in olive AZ and mature-fruit abscission.

### Sphingolipid LCB Content and Composition of Olive AZ during Mature-Fruit Abscission

**FIGURE 4:**
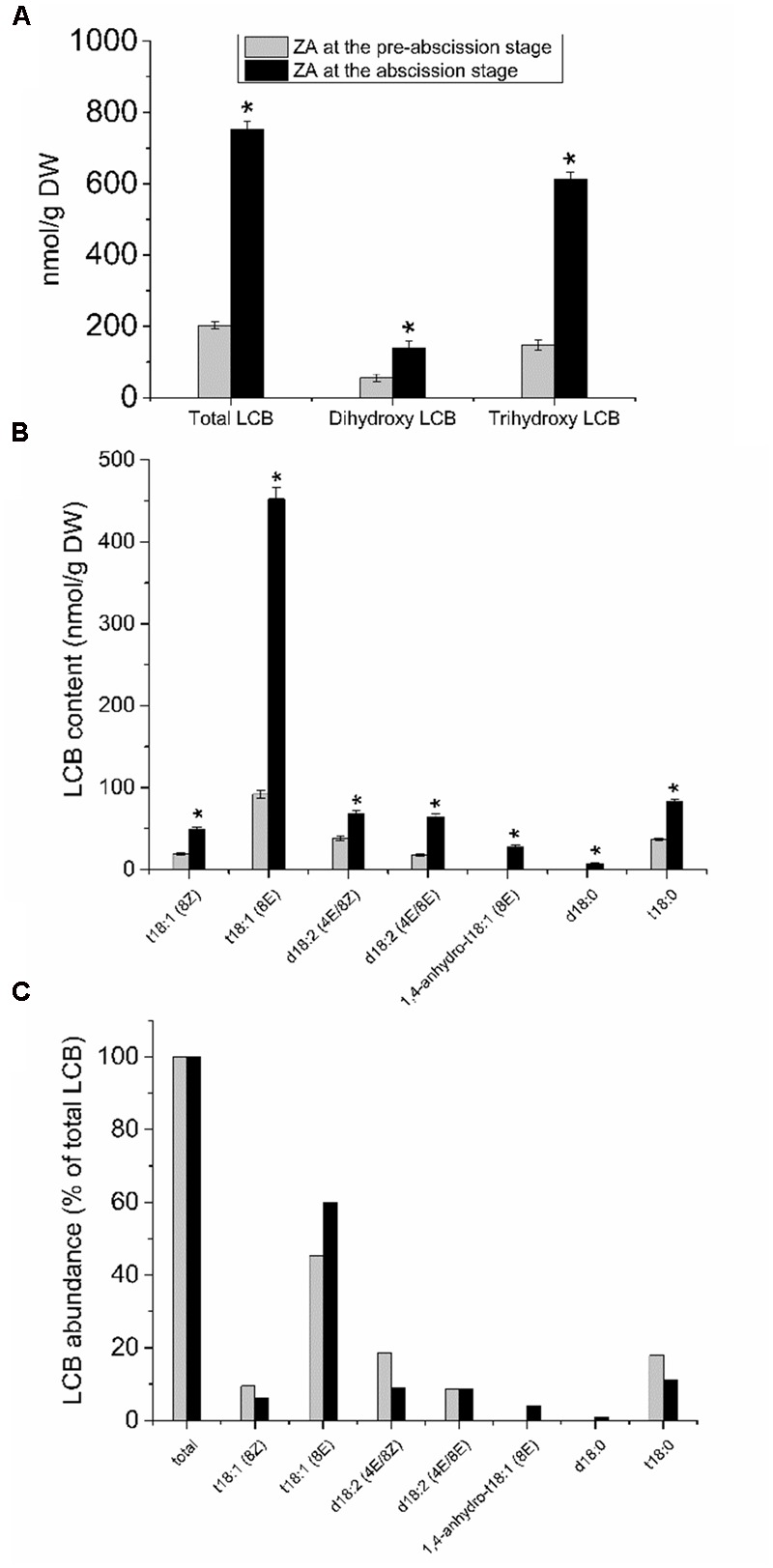
Sphingolipid LCB content and composition of olive AZ during mature-fruit abscission. **(A)** The total LCB content and the relative content of dihydroxy or trihydroxy LCBs in total LCB extracts, **(B)** profiles of LCBs, and **(C)** relative abundance of the different LCB from olive-fruit AZ at two different stages (pre-abscission and abscission). C-The content of every LCB species detected is expressed as a percentage of the total LCB content. Data are the means of three independent biological repeats. Statistically significant differences based on unpaired Student’s *t*-test at *P* < 0.05 are denoted by asterisk. t18:1(Z)-Glc, t18:1(E)-Glc, d18:2(4E/8Z)-Glc, d18:2(4E/8E)-Glc, d18:1(8Z)-Glc, d18:1(8E)-Glc, d18:1(8Z), d18:1(8E), d18:1(4E), d18:2(4E/8E), 1,4-anhydro-t18:1(8Z) and 1,4-anhydro-t18:0 were undetectable in both stages of the ZA.

To investigate the possible relationship between sphingolipid composition and the mature-fruit abscission, we analyzed the LCB profiles derived from hydrolysis of complex sphingolipids in the fruit AZ during abscission. The whole fruit AZ and not their isolated plasma membrane fractions were used for sphingolipid extraction. This was due to the small amounts of samples (the size of the fruit AZ is 1 mm^3^), making it extremely difficult to fractionate the subcellular parts of AZ. As shown in **Figure 4A**, total LCB hydrolyzed from sphingolipids were clearly higher in the olive AZ at the abscission stage as compared to the olive AZ at the pre-abscission stage. A detailed analysis of the total LCB composition showed that, similarly, mature-fruit abscission also significantly raised its dihydroxy and trihydroxy LCB levels in the AZ at the abscission stage (**Figure 4A**), but as ≥81% of the sphingolipid LCBs are trihydroxylated in olive AZ at the abscission stage. In fact, mature-fruit abscission showed an increase in all LCB species studied in olive AZ, t18:1(8E) isomer being the most abundant LCB in olive AZ at the abscission stage (**Figures 4B,C**). This suggests that C-4 hydroxylation and Δ8 desaturation with a preference for (*E*)-isomer formation are quantitatively the most important structural features of the complex sphingolipids present in olive AZ during abscission. In fact, ≥87% of sphingolipid LCBs contained a *cis* or *trans* Δ8 double bond in olive AZ at the abscission stage. On the other hand, we also found that the amount of dihydroxylated LCBs, specially (4*E*, 8*E*)- and (4*E*, 8*Z*)-sphing-4,8-dienine, increased 1.8- and 3.7-fold, respectively, during abscission (**Figures 4B,C**), indicating that dihydroxy LCBs and LCB Δ4 desaturation were found at high levels in olive AZ during abscission. Likewise, in olive AZ, dihydroxylated LCBs such as d18:0 were absent at the pre-abscission stage but were also enriched during abscission (**Figures 4B,C**), suggesting a specific association between these lipids and the mature-fruit abscission in olive. Thus, mature-fruit abscission resulted in quantitative and qualitative changes in sphingolipids in olive AZ.

### Vesicle Trafficking

**FIGURE 5:**
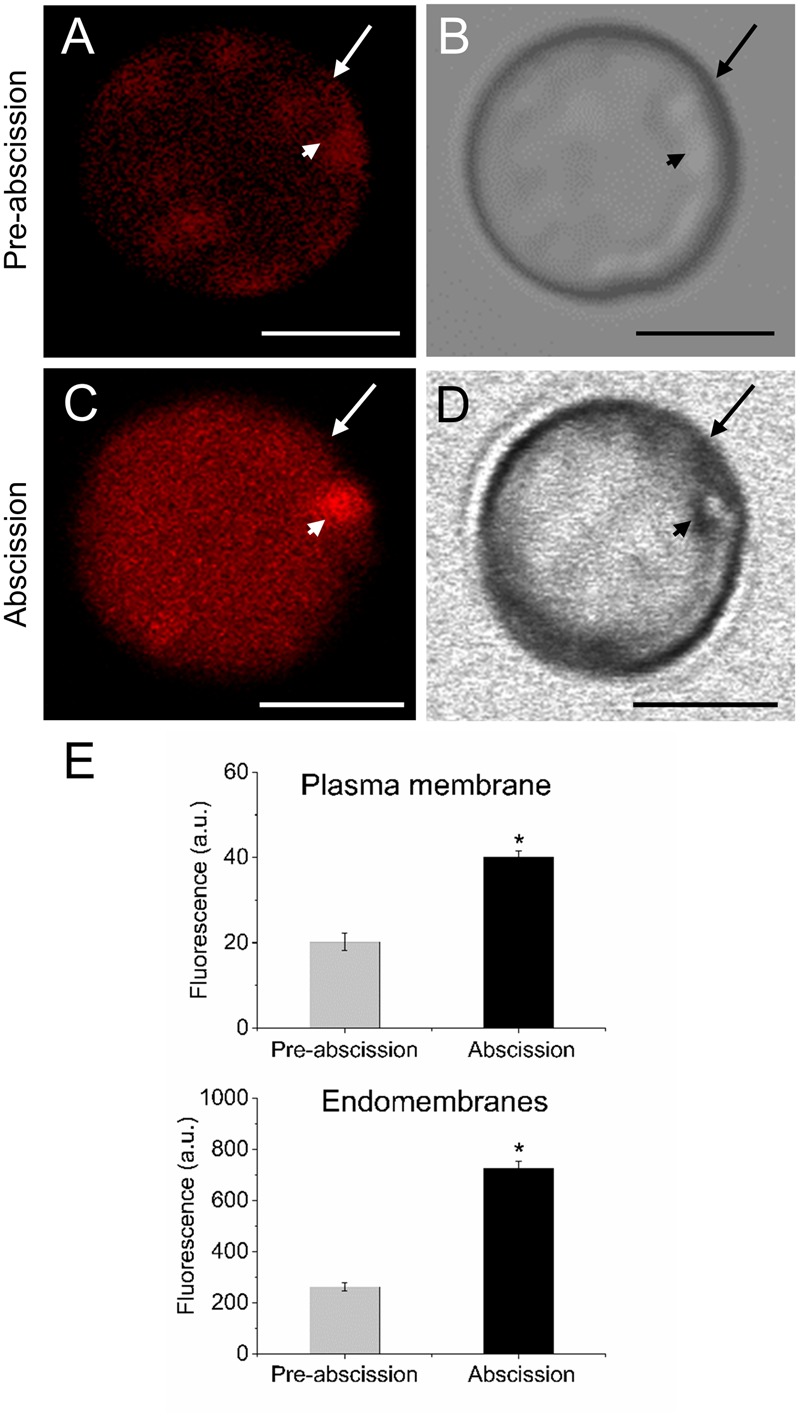
Mature-fruit abscission caused stimulated vesicle trafficking in olive AZ. **(A,C)** Vesicle trafficking was enhanced in fruit AZ at the abscission stage compared with AZ at the pre-abscission stage. **(B,D)** White-light image from fruit AZ protoplast at both stages. Fruit AZ protoplasts stained with FM4-64 were observed under a confocal microscope. **(E)** Relative FM4-64 internalization fluorescence intensity in the plasma membrane (arrow) and the intracellular membranes (arrowhead) in AZ protoplasts at two different stages. About 10 protoplasts were acquired per sample, and data were obtained with FV10 4.2 software (Olympus). Values are means ± SD (*n* = 10 protoplasts). Scale bars are 5 μm. Arrow: plasma membrane. Arrowhead: endomembranes. Statistically significant differences based on unpaired Student’s *t*-test at *P* < 0.05 are denoted by an asterisk.

Because shingolipids have been reported to be intimately involved in endomembrane trafficking (Markham et al., 2013) and the mature-fruit abscission is closely related to endocytosis and exocytosis (Corbacho et al., 2013), we analyzed the vesicle trafficking in olive AZ protoplasts during fruit abscission. Fruit AZs were stained with FM4-64, a reliable styryl dye for membrane trafficking in plant cells (Bolte et al., 2004; Blachutzik et al., 2012). As shown in **Figures 5A,B** the FM4-64 dye was internalized and few fluorescent vesicles were observed in the olive AZ cells at the pre-abscission stage. Conversely, substantial numbers of punctuated fluorescent vesicles were detected in the cytosol of AZ cells at the abscission stage (**Figures 5C,D**). Quantification of FM4-64 showed a significantly increased uptake of FM4-64 in olive AZ cells at the abscission stage compared with olive AZ cells at the pre-abscission stage (**Figure 5E**), indicating that endocytosis is stimulated in the olive AZ during mature-fruit abscission.

### Changes in Squalene and Sterols Associated with Olive Mature-Fruit Abscission

Since sterols are involved in membrane trafficking and some gene expression related to sterol metabolism were up-regulated in the olive AZ during mature-fruit abscission (Gil-Amado and Gomez-Jimenez, 2013), we addressed the possibility that these lipids were involved in the mature-fruit abscission. For this, we extracted total sterols from the olive fruit AZ during abscission. As shown in **Table 1**, squalene, campesterol, and β-sitosterol levels rose in the olive AZ during mature-fruit abscission. By contrast, cycloartenol levels fell during abscission, whereas cholesterol, stigmasterol, and 24-methylene-cycloartenol was not detected in any olive AZ samples (**Table 1**). Thus, measurements of endogenous sterol levels in the olive AZ revealed that β-sitosterol and campesterol are accumulated during mature-fruit abscission.

**Table 1:** Changes in the total sterol content (mg Kg^-1^) in olive AZ during mature-fruit abscission.

mg Kg^-1^	AZ at the pre-abscission stage	AZ at the abscission stage
Squalene	nd	113 ± 7.5
Cycloartenol	725 ± 17.9	527 ± 12.6^∗^
Campesterol	nd	281 ± 16.3
β-Sitosterol	3564 ± 103.1	5323 ± 97.5^∗^
Cholesterol	nd	nd
Stigmasterol	nd	nd
24-Methylene-cycloartenol	nd	nd

*Each value represents the mean of three independent experiments ± SE. Statistically significant differences based on unpaired Student’s *t-*test at *P* < 0.05 are denoted by asterisk; nd, not detectable.*

### Sterol Biosynthetic Gene Expression during Mature-Fruit Abscission

**FIGURE 6:**
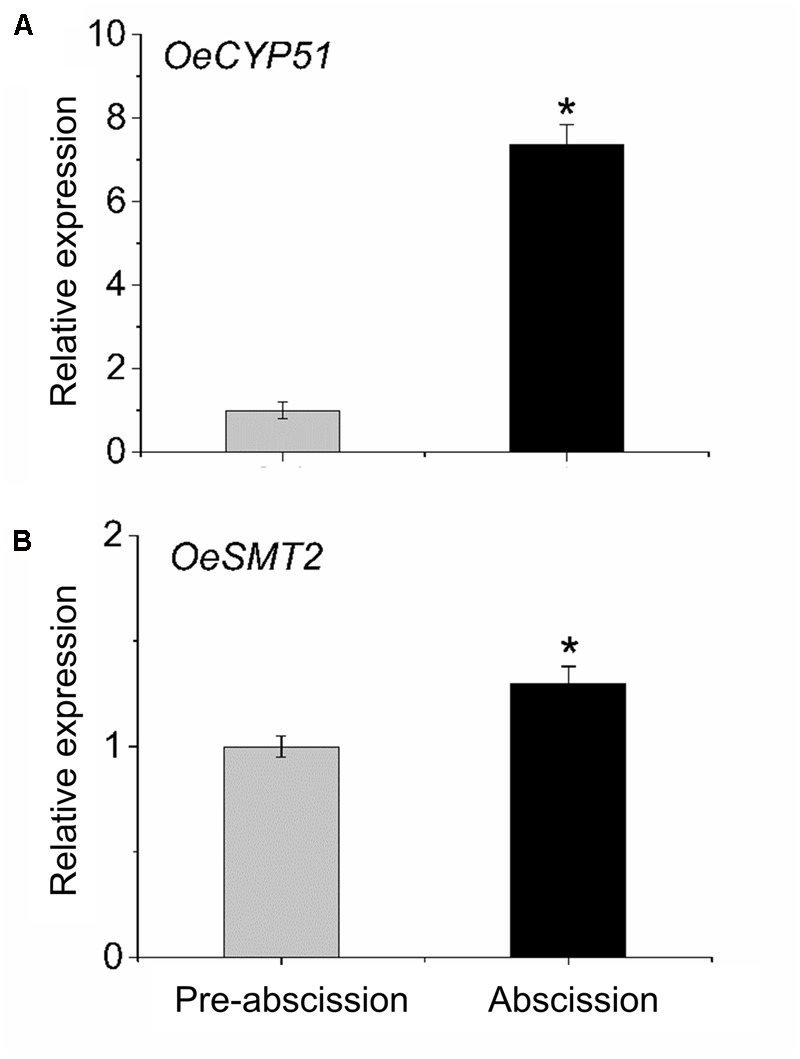
Transcript analysis by real-time PCR of **(A)** *OeCYP51* and **(B)** *OeSMT2* mRNAs in olive AZ during mature-fruit abscission. Total RNAs were isolated from olive-fruit AZs at two different stages (pre-abscission and abscission) and subjected to *q*RT-PCR analysis using *Olea europaea* ubiquitine as an internal control. The data represent the mean values (± SE) of duplicate experiments from three independent biological samples. Statistically significant differences based on unpaired Student’s *t*-test at *P* < 0.05 are denoted by asterisk.

To gain information concerning sterol dynamics during mature-fruit abscission, we addressed the possibility that sterol biosynthetic genes were differentially regulated in the olive AZ during abscission. We assessed the expression profiles of *OeCYP51* and *OeSMT2* genes in fruit AZ during abscission using real-time RT-PCR (**Figure 6**). Olive AZ exhibited a rise in the transcript levels of the *OeCYP51* and *OeSMT2* mRNAs in association with mature-fruit abscission. Thus, *OeCYP51* and *OeSMT2* were up-regulated during mature-fruit abscission parallel to the β-sitosterol content increase.

## Discussion

Plant sphingolipids are structurally diverse molecules that are important as membrane components and bioactive molecules, and participate in an array of processes and environmental responses (Markham et al., 2013; Saucedo-García et al., 2015; Michaelson et al., 2016). To date, sphingolipids have not been taken into consideration in studies dealing with abscission processes. Based on the expression changes of genes involved in sphingolipid biosynthesis and turnover in the olive AZ during mature-fruit abscission (Gil-Amado and Gomez-Jimenez, 2013), the possibility that sphingolipids were involved in the abscission processes was explored.

In the present study, by employing dye, fluorescence labeling, and confocal fluorescence microscopic image analysis, we report changes in the polar lipid and sphingolipid levels and distribution, as well as the distribution of secretory vesicles in live protoplasts from olive AZ cells during mature-fruit abscission. In addition, here, we discuss the sphingolipid LCB and sterol composition changes and their possible relationships during mature-fruit abscission.

Serious technical difficulties complicate the study of the function of individual or bulk lipids in the cell membranes. Lipid imaging is one of the most recent alternative techniques to study membrane lipids, their distribution and localization (Maier et al., 2002; Klymchenko and Kreder, 2014). Unfortunately, an additional limit on these studies in plant membranes is the availability of lipid probes with a structure analogous to that of lipids found in plants (Blachutzik et al., 2012). Therefore, the use of fluorescent lipid analogous to animal cells can help to solve this problem. For example, BD-SM has been developed to monitor the sphingomyelin in animal cells. The rationale for using this lipid in plants is that it can intercalate in any lipid bilayer due to its amphiphilic nature, as has been demonstrated in model membranes for this and other lipids, labeled or not (Klymchenko and Kreder, 2014). In fact, BD-SM has been successfully used in protoplasts from Arabidopsis (Blachutzik et al., 2012). In the present work, the same protocol was used and the fact that protoplasts showed complete exclusion from trypan blue in the presence of the BD-SM indicates that the probe integrates well into the cell membrane without disturbing permeability. This is to be expected, since this fluorescent analog contains a hydrophobic component consisting of sphingosine and a C12-fatty acid, species detected in plants (Markham and Jaworski, 2007). The polar head contains phosphocholine, a polar substituent of phosphatydylcholine, which is another abundant component of plant membranes.

The ability to visualize sphingolipids at the subcellular level is crucial to understand sphingolipid distribution and function in plant cells. In the present study, an increase in the relative content of polar lipids and sphingolipids was positively correlated with mature-fruit abscission in live protoplasts from olive AZ, suggesting that sphingolipids can participate as structural and/or signaling molecules during abscission. Previous data from RNA-Seq (Gil-Amado and Gomez-Jimenez, 2013) indicated expression of sphingolipid biosynthesis-related genes at higher levels in olive AZ during abscission. These included one serine palmitoyltransferase (SPT; Uniprot ID: B9RWA7), one 3-ketoacyl-CoA synthase 4 (KCS4, VLCFA 4, At1g19440; Uniprot ID: Q9LN49) as well as one acyl-CoA-independent ceramide synthase (AtCES1, At4g22330; Uniprot ID: Q94IB9), two alkaline phytoceramidases (CDases; Uniprot ID: D7SZU8 and B9RXD0), one CDase (Uniprot ID: B9SPF3) and a glucosylceramidase (GlcCerase; UniProt ID: B9RWK0). Complex sphingolipids in plants are formed by a ceramide backbone comprised of a LCB esterified to a long-chain fatty acid (long-chain FA or VLCFA) (Michaelson et al., 2016). Evidence indicates that free LCBs or ceramides are second messengers in programmed cell death (PCD) in plant cells (Pata et al., 2010; Michaelson et al., 2016), though their precise role in abscission remains unknown. During abscission in tomato, PCD reportedly occurs asymmetrically (Bar-Dror et al., 2011), casting doubts as to whether ceramides and LCBs can act as molecules in the olive AZ to promote PCD. Notably, in our pyrosequencing data set, we include genes that encode enzymes for sphingolipid turnover among the up-regulated genes (Gil-Amado and Gomez-Jimenez, 2013). Also highly expressed during abscission in the olive AZ is sequence that encodes a sphingosine phosphate lyase (SPL; Uniprot ID: B9RMB8), involved in sphingosine hydrolysis (Gil-Amado and Gomez-Jimenez, 2013). Thus that sphingolipid turnover during mature-fruit abscission can be considered an active process or the regulation of LCB-P levels may have some physiological significance in the olive AZ during the abscission of mature fruit. Thus, the results of our studies show a relationship between sphingolipid content/biosynthetic gene expression and mature-fruit abscission in olive, suggesting that a complex regulatory network of sphingolipid metabolism exists in AZ during abscission.

Plants contain a large diversity of sphingolipid structures, arising in part from C4 hydroxylation and Δ4 and Δ8 desaturation of the LCB moiety. As we know that SPT is the enzyme that catalyses the first reaction in sphingolipid metabolism and one *SPT* and one *SPL* gene was up-regulated during abscission (Gil-Amado and Gomez-Jimenez, 2013), we hypothesized that mature-fruit abscission caused a general rise in LCBs levels, especially of sphinganine (d18:0). Sphinganine is the first LCB synthesized in this pathway and is a substrate for desaturases and hydroxylases to generate the other LCBs (Chen et al., 2009). The profiling of sphingolipid LCB measurement revealed that both the di- and trihydroxylated LCBs increased in the olive AZ during mature-fruit abscission. The sphingolipids with the trihydroxylated LCB (*E*)-phytosphingo-8-enine (t18:1^8^*^E^*), which is more abundant, increased 4.9-fold, with (*Z*)-phytosphingo-8-enine (t18:1^8^*^Z^*) increased 2.5-fold, and with phytosphingosine (t18:0) increased 2.3-fold in the olive AZ at the abscission stage compared to the pre-abscission stage. Thus, in the present study, we revealed that sphingolipid with C-4 hydroxylation and Δ8 desaturation with a preference for (*E*)-isomer formation are quantitatively the most important sphingolipids in olive AZ during abscission, suggesting that LCB C4 hydroxylation and Δ8 desaturation could be important in mediating total sphingolipid amounts or levels of specific sphingolipid classes in olive AZ during abscission. In particular, ≥81% of the sphingolipid LCBs are trihydroxylated and ≥87% of sphingolipid LCBs contain a *cis* or *trans* Δ8 double bond in olive AZs at the abscission stage. These data fit with those in most organs of Arabidopsis where 90% of all LCBs contain a *cis* or *trans* Δ8 monounsaturation (Markham et al., 2006). In Arabidopsis, LCB Δ8 desaturation contributes to the structural properties of the plasma membrane influencing the tolerance to environmental extreme conditions (da Silva et al., 2006; Ryan et al., 2007; Chen et al., 2012) These desaturated species are distributed to ceramides of the two major sphingolipid classes, glucosylceramides (GlcCers) and glycosyl inositol phosphoceramides (GIPCs): GlcCer synthesis takes place in the ER and GIPC synthesis in the Golgi (Chen et al., 2012). In plants, GlcCers and GIPCs have distinctive ceramide compositions: GlcCers are enriched in dihydroxy LCBs and C16 fatty acids whereas GIPCs are enriched in trihydroxy LCBs and VLCFAs (Sperling et al., 2005; Markham et al., 2006, 2013). In the present study, LCB content and composition analysis derived from sphingolipid hydrolysis demonstrated an increase of trihydroxylated LCBs in the olive AZ during abscission, suggesting an enrichment of sphingolipids that contain these LCB forms and VLCFA into their backbones. Our results indicate that GIPCs are more abundant than GlcCers in the olive AZ during abscission. This agrees with our previous results in which we demonstrated the up-regulation of two 3-keto-acyl-CoA synthases genes, *KCS2* and *KCS11*, in the olive AZ during abscission (Gil-Amado and Gomez-Jimenez, 2013), suggesting an increase of VLCFAs in olive AZ during abscission and thus a correlation between the mature-fruit abscission and the substrate specificity based primarily on chain length (C20-C22) as well as with the characteristics of the VLCFA lipid pool downstream (DAISY and suberin). Overall, these data are consistent with a possible increased VLCFA biosynthetic capacity in the AZ, as suggested by sphingolipid LCB profiling.

Recently, it was demonstrated that the length of the ceramide acyl chain is determinant for sphingolipid function. Sphingolipids with VLCFA are critical for plant growth and in the secretory pathway for specific cargo proteins (Markham et al., 2011). In addition, FB1-treated BY2 cells with affectation on sphingolipid content and composition showed alterations in the exocytosis pathways but minor changes at endocytosis routes. FB1 causes an inhibition of ER to GA transport of cargo molecules, as in yeast and animal cells (Aubert et al., 2011). The VLCFA are necessary for sorting specific membrane vesicles from the TNG toward the plasma membrane. During mature-fruit abscission, the trafficking and secretion of enzymes is a very active process (Corbacho et al., 2013). Secretion of cell-wall hydrolytic enzymes such as polygalacturonases, cellulases, expansines and others alter cell walls and dissolve the pectin-rich middle lamella in the AZ (Liljegren et al., 2009; Corbacho et al., 2013; Gil-Amado and Gomez-Jimenez, 2013). TGN is a compartment where endocytosis/recycling is also present. During mature-fruit abscission, endocytosis has been postulated to internalize cell-wall materials for recycling or degradation (Corbacho et al., 2013). In the present study, our data reveal that endocytosis, visualized by staining with fluorescent dye FM4-64, was strongly stimulated in AZ during mature-fruit abscission with a concomitant increase in sphingolipid content. Previously, our pyrosequencing data also indicated higher olive AZ expression of vesicle trafficking-related genes for the small GTPases, dynamins, V-type ATPases, reticulons, and syntaxins during abscission (Gil-Amado and Gomez-Jimenez, 2013). In particular, seven *RAB-GTPase* genes, *RABG3A, RABG3D, ARA4, ARA6, RABA2B, RABA2A/RAB11C* and *RABC2A*, were up-regulated in olive AZ during mature-fruit abscission (Gil-Amado and Gomez-Jimenez, 2013). Overall, these data are consistent with an increased endomembrane trafficking in olive AZ during abscission, as indicated by FM4-64 staining, suggesting that endomembrane trafficking probably modulates cell wall modifications during olive mature-fruit abscission.

Interestingly, sterols that are also extensively involved in endocytosis, increased their content in the olive AZ during mature-fruit abscission. Although plant sterols participate in many physiological processes (Clouse, 2002; Souter et al., 2002; He et al., 2003; Schaller, 2003; Men et al., 2008; Kim et al., 2010; Schrick et al., 2012; Qian et al., 2013; Li et al., 2015; Valitova et al., 2016) their potential role in abscission processes remains to be determined. Measurements of endogenous sterol levels in the olive AZ revealed that the proportions of β-sitosterol, and campesterol were higher during mature-fruit abscission and with no detection of cholesterol or stigmasterol. The regulation of the ratios between the different types of sterols and of sterols/sphingolipids can be of crucial importance in the responses of plants to stress (Valitova et al., 2016). The main enzymes involved in plant sterol biosynthesis are 3-hydroxy-3-methylglutaryl CoA reductase, sterol-C24-methyltransferase (SMT), and C22 sterol desaturase. These enzymes are responsible for maintaining the optimal balance between sterols. In addition, in the present study, our data revealed the up-regulation of genes encoding an obtusifoliol 14α-demethylase involved in the postsqualene sterol biosynthetic pathway (*CYP51)* and a SMT2 in olive AZ during mature-fruit abscission, verifying our pyrosequencing data (Gil-Amado and Gomez-Jimenez, 2013) as well as the contention that these transcripts are enriched in the AZ throughout abscission. Hence, in the AZ of olive, cycloartenol is converted into functional phytosterols is induced during abscission, this being induced by the up-regulation of genes that encode SMT2 and CYP51. This appears to signify that SMT2 up-regulation may directly regulate the ethyl/methyl end-product sterol ratio (sitosterol:campesterol) in the olive AZ during abscission. Overall, these data are consistent with an increased β-sitosterol biosynthetic capacity in olive AZ during abscission, as indicated by sterol profiling. Therefore, the strong induction of sphingolipid/sterol levels as well as the high expression of the genes related to sphingolipid and sterol synthesis in the olive AZ, might indicate that both lipid classes function in the cell-surface activities at least for protein trafficking to the plasma membrane during abscission. The involvement of sterols and sphingolipids in the formation of membrane nanodomains must be considered. These membrane regions could be constituting signaling platforms containing signaling proteins, such as ion channel proteins, receptor kinases, small GTPases, and Ca^2+^-regulated proteins, enriched in membrane microdomains (Chen et al., 2009) and up-regulated during mature-fruit abscission in olive AZ (Gil-Amado and Gomez-Jimenez, 2013). Therefore, our results using microscopy combined with biochemical and molecular approaches provide new insights into the functions of sphingolipids and sterols in plants.

## Conclusion

Taken together, our results demonstrate that lipid content of the olive AZ change both qualitatively and quantitatively during mature-fruit abscission. These data show large differences in sphingolipid and sterol content/composition and biosynthetic gene expression in olive AZ during abscission, including the identification of specific LCB and sterol in AZ during abscission, indicating that these sphingolipids and sterols may play a role in AZ during mature-fruit abscission.

## Author Contributions

MP-L performed the experiments. MP and JL sampled the material and contributed to sphingolipid and sterol analysis. MS-G and MG-R contributed to analyze and discuss the results and prepare the manuscript. MG-J conceived and supervised the study, wrote and critically revised the manuscript.

## Conflict of Interest Statement

The authors declare that the research was conducted in the absence of any commercial or financial relationships that could be construed as a potential conflict of interest.

## Supplementary Material

FIGURE S1 Changes on non-polar/polar lipid ratio of live protoplasts from olive AZ during mature-fruit abscission. Fluorescence of variable colors from protoplast indicating difference in neutral lipid composition: polar lipid appeared as red **(A,E)** and neutral or non-polar lipid appeared as green **(B,F)**. **(A,B)** Nile red stained protoplasts from the olive AZ at the pre-abscission stage compared to the white-light image **(C)**. **(E,F)** Nile red stained protoplasts from the olive AZ at the abscission stage compared to the white-light image **(G). (D,H)** Viability was successfully tested using trypan blue. Scale bars are 5 μm. **(I)** Effect of the abscission on non-polar:polar lipid ratio of fruit AZ protoplasts. Columns and bars indicate means ± SD, respectively, from five independent experiments. Statistically significant differences based on unpaired Student’s *t*-test at *P* < 0.05 are denoted by asterisk.
