# Supplementary figures and images for: Localization of Sphingolipid Enriched Plasma Membrane Regions and Long-Chain Base Composition during Mature-Fruit Abscission in Olive
Source: Front Plant Sci. 2017 Jun 29;8:1138. doi: 10.3389/fpls.2017.01138 (PMC5489598; doi:10.3389/fpls.2017.01138)

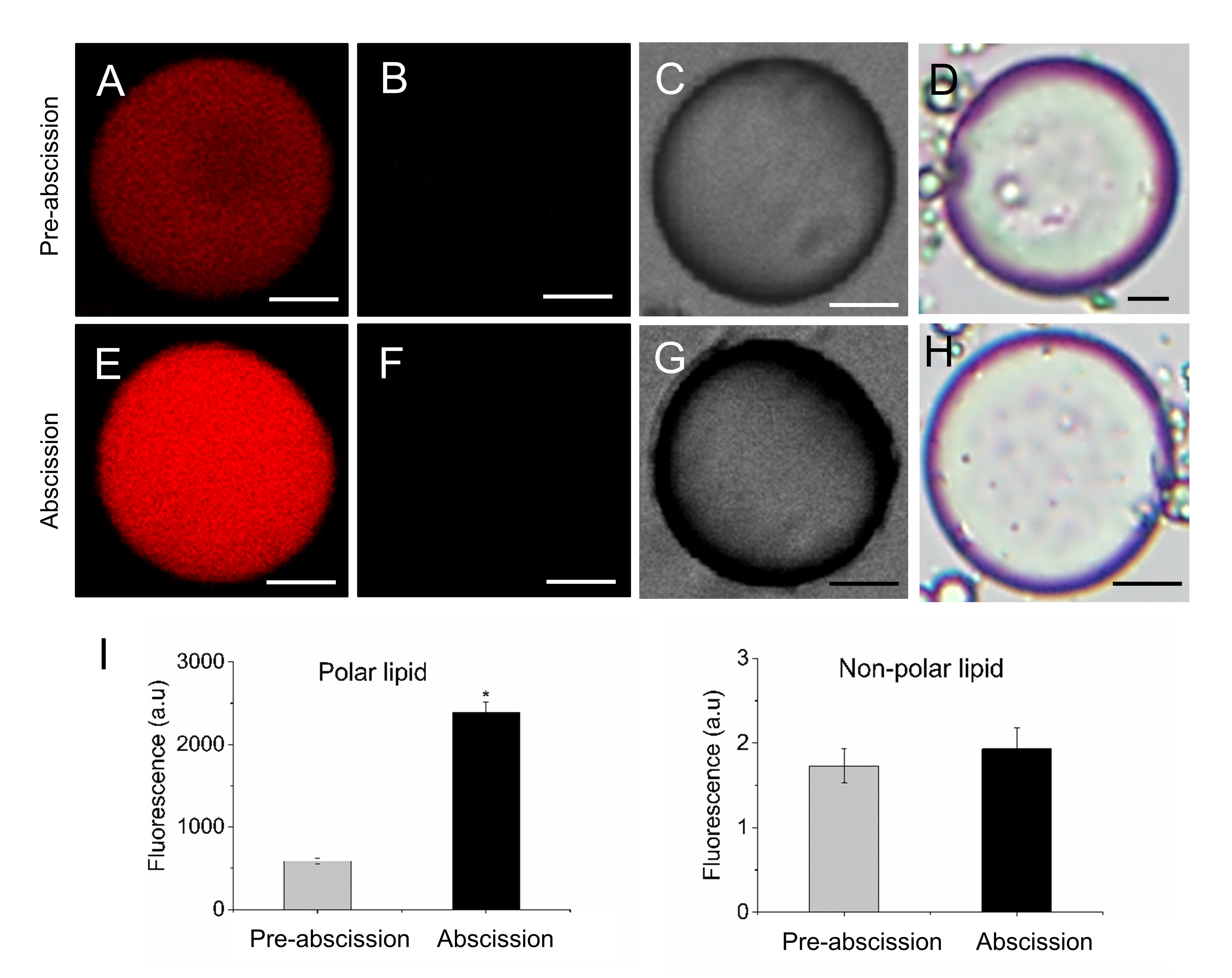

Supplement: FIGURE S1 — Changes on non-polar/polar lipid ratio of live protoplasts from olive AZ during mature-fruit abscission. Fluorescence of variable colors from protoplast indicating difference in neutral lipid composition: polar lipid appeared as red (A,E) and neutral or non-polar lipid appeared as green (B,F). (A,B) Nile red stained protoplasts from the olive AZ at the pre-abscission stage compared to the white-light image (C). (E,F) Nile red stained protoplasts from the olive AZ at the abscission stage compared to the white-light image (G). (D,H) Viability was successfully tested using trypan blue. Scale bars are 5 μm. (I) Effect of the abscission on non-polar:polar lipid ratio of fruit AZ protoplasts. Columns and bars indicate means ± SD, respectively, from five independent experiments. Statistically significant differences based on unpaired Student’s t-test at P < 0.05 are denoted by asterisk. [file Image_1.TIF]
